# Propeller Flaps in Extremity Sarcoma Reconstruction: A Systematic Review of Reconstructive Outcomes and Oncologic Considerations

**DOI:** 10.3390/curroncol33050269

**Published:** 2026-05-06

**Authors:** Sara Matarazzo, Beatrice Corsini, Claudia Lauricella, Elisa Bascialla, Luigi Valdatta, Ferruccio Paganini

**Affiliations:** 1 Division of Plastic and Reconstructive Surgery, Department of Biotechnology and Life Sciences, University of Insubria, 21100 Varese, Italy; 2 Division of Plastic Surgery, IRCCS European Institute of Oncology, 20141 Milan, Italy; 3 Department of Medicine and Surgery, University of Insubria, 21100 Varese, Italy

**Keywords:** soft tissue sarcoma, propeller flap, perforator flap, extremity reconstruction, limb salvage, oncologic reconstruction, perforator-based propeller flap, lower limb reconstruction, reconstructive surgery

## Abstract

Extremity soft tissue sarcoma resection often results in complex defects requiring reconstruction to preserve function and support multidisciplinary treatment. Propeller flaps have emerged as a local alternative to free flaps in selected cases, but their role in sarcoma reconstruction remains incompletely defined. This systematic review aimed to assess the current evidence on the indications, reconstructive outcomes, and oncologic reporting of propeller flaps in extremity sarcoma reconstruction. A systematic literature search was conducted in PubMed/MEDLINE, Scopus, and the Cochrane Library in accordance with PRISMA 2020. Studies reporting perforator-based propeller flaps after extremity soft tissue sarcoma resection were included. Data were synthesized descriptively because of the heterogeneity of study design, patient populations, and outcome reporting. Nineteen studies were included. Across the published literature, 656 patients were described overall, including 185 propeller flaps used after sarcoma resection. Most reconstructions involved the lower extremity, accounting for more than 95% of reported cases. Flap survival was generally high; among studies providing extractable numerical data, complete flap survival was 92.7% (115/124), total flap loss was 3.8% (2/52), and partial necrosis occurred in 9.6% (5/52) of flaps. Venous congestion was the most frequently reported complication. Oncologic outcomes were inconsistently reported, and comparative recurrence-related data were very limited. Available data suggest that propeller flaps can provide reliable coverage with acceptable complication rates in selected cases. Oncologic outcomes were sparsely and inconsistently reported, and the current literature does not show a clear signal of increased local recurrence; however, the available evidence is insufficient to draw firm conclusions regarding oncologic safety or equivalence compared with other reconstructive strategies. These findings support consideration of propeller flaps as a complementary reconstructive option in carefully selected patients, although higher-quality studies with standardized oncologic outcomes are needed.

## Simple Summary

Resection of extremity soft tissue sarcomas often creates complex defects that require reconstruction to preserve limb function and support oncologic treatment. Microsurgical free flaps are widely used for this purpose but involve longer and more technically demanding procedures. Propeller flaps, which use nearby perforator-based tissue, have emerged as a local reconstructive option in selected cases. This systematic review summarizes the available literature on propeller flaps in extremity sarcoma reconstruction. The published evidence suggests that these flaps may provide satisfactory reconstructive outcomes in carefully selected patients and may broaden the reconstructive options available in this setting. However, the available literature remains limited, and further studies are needed to better define their role.

## 1. Introduction

Soft tissue sarcomas (STSs) of the extremities represent a rare and heterogeneous group of malignancies that require a multidisciplinary approach, combining oncologic resection and functional reconstruction [1,2,3,4]. Limb-sparing surgery has progressively replaced amputation as the standard of care, leading to an increasing demand for reliable reconstructive strategies capable of restoring soft tissue coverage while preserving limb function and enabling timely adjuvant therapy [1,5,6,7]. However, wide oncologic excision frequently results in complex defects characterized by exposed bone, tendons, joints, or neurovascular structures, posing significant reconstructive challenges [8,9,10].

Traditionally, microsurgical free flaps have been considered the gold standard for the reconstruction of large post-sarcoma defects, offering ample tissue availability and reliable vascularity [11,12]. Nonetheless, free tissue transfer is associated with longer operative time, eventual donor-site morbidity, and the need for microsurgical expertise and suitable recipient vessels, which may be compromised in previously irradiated or scarred tissues [4,13,14,15,16,17,18]. Conversely, local random-pattern flaps are often inadequate in the oncologic setting due to their limited arc of rotation, less reliable vascularity, and limited capacity to cover the large defects typically resulting from tumor resection [19,20,21].

**Figure 1:**
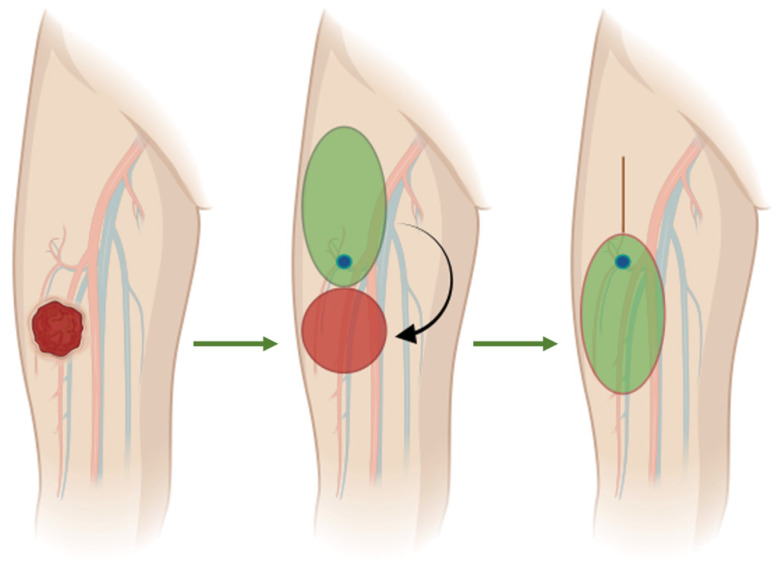
Schematic illustration of perforator-based propeller flap reconstruction for a thigh defect after sarcoma excision. The left panel shows the localization of the thigh sarcoma. The middle panel illustrates the post-excisional defect (red), the planned propeller flap (green), and the selected perforator (blue). The right panel shows the final reconstruction obtained after 180° rotation of the flap around its pivot point, corresponding to the perforator vessel.

In this context, perforator-based propeller flaps have emerged as a versatile alternative, allowing the transfer of well-vascularized tissue based on a perforator with minimal donor-site morbidity [21,22,23,24]. Since their conceptualization and subsequent popularization over the past two decades, propeller flaps have been increasingly adopted in reconstructive surgery for trauma, chronic wounds, and oncologic defects. Their ability to provide “like-with-like” tissue, avoid microvascular anastomosis, and preserve major vascular axes makes them particularly appealing in selected extremity reconstructions [25,26,27] (Figure 1).

Despite their growing use, the role of propeller flaps in extremity sarcoma reconstruction remains ill-defined. In current practice, these flaps appear most suitable for carefully selected patients with moderate-sized defects, favorable perforator anatomy, and reconstructive needs that can be adequately addressed with local tissue transfer without clearly requiring microsurgical free flap reconstruction. Their potential value is particularly relevant when a local perforator-based solution may provide stable coverage while limiting operative complexity and donor-site morbidity. However, the available literature remains limited, often based on small heterogeneous series, and does not yet clearly define the boundaries of their indication in oncologic limb reconstruction. Moreover, concerns persist regarding their oncologic safety, particularly in relation to reconstruction within previously irradiated fields and the potential proximity of transferred tissues to tumor margins [28]. These uncertainties contribute to variability in clinical practice and may partially explain the cautious adoption of propeller flaps in oncologic limb reconstruction.

Therefore, the aim of this study was to systematically review the current literature on propeller flaps in extremity sarcoma reconstruction, focusing on their indications, reconstructive outcomes, and the limited available oncologic evidence. By critically appraising the published data, we sought to clarify the current role of propeller flaps within the reconstructive algorithm and to identify the main gaps that should be addressed by future research.

## 2. Materials and Methods

### 2.1. Search Strategy

**Figure 2:**
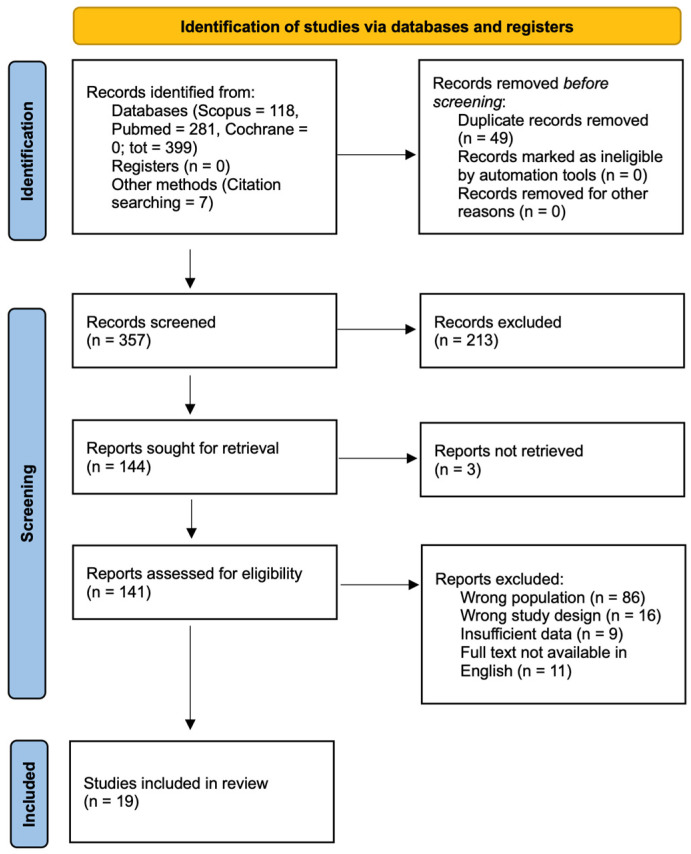
PRISMA 2020 flow diagram.

A comprehensive literature search was conducted on 30 November 2025, in PubMed/MEDLINE, Scopus, and the Cochrane Library, in accordance with the Preferred Reporting Items for Systematic Reviews and Meta-Analyses (PRISMA) 2020 guidelines [29]. The search strategy was designed to identify studies reporting the use of propeller flaps for reconstruction following extremity soft tissue sarcoma resection. Search terms included multiple variations and related concepts for both the reconstructive technique and the oncologic condition. Keywords related to the reconstructive procedure included terms such as *propeller flap*, *propeller flaps*, *perforator flap*, *perforator flaps*, *perforator-based flap*, *perforator propeller*, and specific perforator-based flap terminology. These were combined with terms related to the oncologic context, including *sarcoma*, *soft tissue sarcoma*, *soft tissue tumor*, *soft tissue tumour*, *soft tissue neoplasm*, *malignant tumor*, and *limb-sparing* or *limb salvage*. Additional anatomical terms referring to extremity reconstruction (e.g., *limb*, *extremity*, *upper limb*, *lower limb*, *arm*, *leg*, *hand*, *foot*, *thigh*, *forearm*, and *ankle*) were also incorporated to increase search sensitivity. Database-specific adaptations of the search strategy were applied to account for differences in indexing systems and search interfaces. The full electronic search strategies for each database, including the complete search strings used, are provided in the Supplementary Materials (File S1) to ensure full reproducibility of the search process. In addition, the reference lists of all included studies were manually screened to identify further relevant publications (backward citation tracking). The PRISMA checklist is provided in the Supplementary Materials (Table S1), and the study selection process is illustrated in the PRISMA flow diagram (Figure 2). This systematic review was registered in PROSPERO (registration number: CRD420261349550).

### 2.2. Eligibility Criteria

Studies were considered eligible if they met the following criteria:

- Original clinical studies, including prospective or retrospective studies, case series, and case reports describing clinical applications;
- Reports describing the use of perforator-based propeller flaps for reconstruction following resection of soft tissue sarcomas of the upper or lower extremities;
- Studies involving human subjects;
- Articles published in English;
- Full-text availability.

Studies were excluded if they met any of the following criteria:

- Cadaveric, anatomical, or experimental animal studies;
- Studies not involving extremity sarcomas (e.g., trunk or head and neck locations);
- Mixed cohorts in which sarcoma-specific outcomes could not be clearly isolated;
- Reviews, editorials, commentaries, expert opinions, or conference abstracts without full-text data;
- Articles lacking a clear description of the propeller flap technique;
- Articles published in languages other than English.

Eligibility was independently assessed by three reviewers according to predefined inclusion criteria. Any discrepancies were resolved through discussion and consensus among the authors. When studies reported heterogeneous etiologies (e.g., trauma, chronic wounds, or other malignancies), only sarcoma-specific data were extracted when available.

### 2.3. Data Extraction

The data extraction process was conducted using a standardized Microsoft Excel spreadsheet v2210 (Microsoft Corporation, Redmond, WA, USA) specifically developed for this review. Three independent reviewers screened the included studies and manually extracted the relevant variables. Any discrepancies were resolved through discussion and consensus. For each study, bibliographic information was recorded, including first author, year of publication, journal, and study design (prospective, retrospective, case series, or case report). Patient and tumor-related variables were collected when available, including the number of patients, number of propeller flaps, and patient age. Particular attention was given to isolating sarcoma-specific data in studies reporting heterogeneous cohorts. Reconstructive characteristics were extracted, including anatomical location (upper or lower extremity), number of propeller flaps, and technical details when provided. In studies reporting mixed reconstructive techniques, only data specifically referring to propeller flaps were analyzed. The oncologic context was carefully assessed. Information regarding sarcoma type, use of neoadjuvant or adjuvant therapies (e.g., radiotherapy or chemotherapy), and reconstructive timing was recorded when available.

### 2.4. Outcomes Definitions

Outcomes were grouped into reconstructive and oncologic domains to reflect the dual nature of extremity sarcoma reconstruction. Reconstructive outcomes included flap survival, flap failure, postoperative complications, and the need for revision procedures. Flap success was defined as complete flap survival without total necrosis, whereas flap failure was defined as total flap loss requiring revision surgery or alternative reconstruction. Partial flap loss and other complications were recorded separately when reported, including venous congestion, wound dehiscence, infection, hematoma, seroma, and partial necrosis.

Oncologic outcomes included local recurrence and disease progression when reported. Additional variables extracted included follow-up duration, need for revision procedures, and functional outcomes such as return to ambulation, recovery of daily activities, and patient-reported satisfaction when available. Any relevant remarks or conclusions provided by the study authors were noted to inform the interpretative analysis.

When outcome definitions differed across studies, the original authors’ definitions were retained and summarized descriptively. When information was not explicitly reported in the original article, the variable was classified as not reported (NR). When studies included mixed reconstructive techniques or heterogeneous etiologies, only data specifically related to propeller flaps in sarcoma patients were extracted and analyzed when available. When outcomes were reported for mixed populations but could not be disaggregated for the sarcoma subgroup, the variable was classified as not available (NA).

When reported, outcomes were described at the flap level, since several studies reported outcomes per flap rather than per patient.

### 2.5. Quality Assessment

The methodological quality of the included studies was assessed using different tools according to study design. Observational studies were evaluated using the National Institutes of Health (NIH) Quality Assessment Tool for Observational Cohort and Cross-Sectional Studies (National Heart, Lung, and Blood Institute, 2014) [30], while case reports and case series were assessed using the Joanna Briggs Institute (JBI) Critical Appraisal Checklists for Case Reports and Case Series [31]. Quality assessment was performed independently by three reviewers, with disagreements resolved through discussion and consensus. Based on the NIH criteria, observational studies were qualitatively judged as good, fair, or poor according to the overall fulfillment of the checklist items and the reviewers’ assessment of methodological rigor (Table 1). Given the exploratory nature of the available evidence and the predominance of case series and small observational studies, quality assessment was used to support the interpretative synthesis rather than to exclude studies a priori.

### 2.6. Data Synthesis

Due to the heterogeneity of study designs, patient populations, reconstructive techniques, and outcome reporting, a formal quantitative meta-analysis was not feasible. Therefore, the available evidence was synthesized descriptively. When sufficient numerical data were available, descriptive estimates were calculated from aggregated extractable patient- or flap-level data reported across studies. These figures do not represent study-level pooled estimates and should be interpreted as descriptive summaries only. Continuous variables were summarized using ranges and measures of central tendency when reported, while categorical variables were described as absolute values and percentages. No formal comparative statistical testing was performed due to the heterogeneity of the included studies and the predominance of small case series. Given the exploratory nature of the available literature, the analysis should be interpreted as a descriptive synthesis of the current evidence rather than as a quantitative analysis.

## 3. Results

The database search identified 399 records (PubMed *n* = 281, Scopus *n* = 118, Cochrane Library *n* = 0), with an additional 7 studies retrieved through manual screening of reference lists, for a total of 406 records. After removal of 49 duplicates, 357 records were screened by title and abstract. A total of 144 articles were selected for full-text assessment. Three reports could not be retrieved, leaving 141 articles assessed for eligibility. Of these, 122 were excluded for the following reasons: wrong population (*n* = 86), inappropriate study design (*n* = 16), insufficient data (*n* = 9), or non-English full text (*n* = 11). Nineteen studies met the inclusion criteria and were included in the final qualitative synthesis (Figure 2). Overall, the methodological quality of the included studies was limited. Most of the available evidence consisted of case reports, small case series, and retrospective observational studies. The main recurrent limitations identified through quality appraisal included small sample size, retrospective design, lack of control groups, heterogeneous outcome reporting, and limited sarcoma-specific data in mixed cohorts. Therefore, the findings of this review should be interpreted cautiously and primarily as descriptive. Whenever numerical synthesis was possible, estimates were derived from aggregated extractable patient- or flap-level data and not all studies contributed to all denominators.

### Descriptive Analysis

Nineteen studies published between 2010 and 2024 were included in the final analysis [32,33,34,35,36,37,38,39,40,41,42,43,44,45,46,47,48,49,50]. The available evidence largely consisted of case reports, small case series, and observational studies. Overall, the literature was characterized by small sample sizes and heterogeneous reporting (Table 1). The main findings reported across the included studies are summarized in Table 2.

Across the included studies, 656 patients were described overall; however, only 185 underwent reconstruction with propeller flaps following sarcoma resection, and a substantial proportion of the available evidence derived from mixed cohorts in which sarcoma-specific data had to be selectively extracted. The mean patient age was 59.8 years (range 11–92). A slight female predominance was observed, with a male-to-female ratio of 0.87. Comorbidities were inconsistently reported; however, among studies providing data, the most frequently described risk factors included diabetes mellitus, smoking history, and peripheral vascular disease. Preoperative radiotherapy was documented in a minority of cases but suggested a population with a relatively high burden of vascular and metabolic comorbidities.

Histologic subtype was variably reported. Among studies providing detailed data, myxofibrosarcoma was the most frequently described entity, followed by undifferentiated pleomorphic sarcoma. Leiomyosarcoma and synovial sarcoma were less commonly reported, while liposarcoma and other rare subtypes were only sporadically described.

Defects were predominantly located in the lower extremity, accounting for more than 95% of cases (178/182). The most commonly involved regions were the thigh and peri-knee area, followed by the pretibial and distal leg regions. Upper extremity involvement was uncommon. Defects were generally moderate to large in size. The maximum diameter most frequently ranged between 10 and 15 cm, and reported defect areas typically ranged from 80 to 160 cm^2^. Small defects (<5 cm) were rarely described. Propeller flaps were consistently designed larger than the corresponding defect. The flap major axis most commonly ranged between 17 and 24 cm, with reported extremes from approximately 10 to nearly 28 cm. When reported, flap area generally ranged from 100 to 220 cm^2^. Most reconstructions employed fasciocutaneous propeller flaps elevated in a subfascial plane, with only rare cases including a muscular component. Immediate reconstruction was the most common approach, while delayed reconstruction was reported in only a small minority of cases. Rotation angles were typically between 90° and 180°, with full 180° rotations accounting for the majority of cases. Reported mean rotation angles ranged from approximately 106° to 160°, with an estimated overall mean of 130–150°.

Flap survival across the included studies was generally high. When sufficient numerical data were available, reconstructive outcomes were summarized at the flap level by aggregating the number of reported events across studies relative to the total number of reconstructed flaps. Among the extractable flap-level data reported in the included studies, complete flap survival was 92.7% (115/124), total flap loss was 3.8% (2/52), and partial necrosis occurred in 9.6% of flaps (5/52), with reported rates ranging between approximately 5% and 20%, depending on the definitions used by individual studies. These figures were derived from aggregated extractable data and should be interpreted as descriptive estimates rather than formal pooled results. Venous congestion emerged as the most commonly reported mechanism of flap compromise. Other complications were sporadically reported and included wound dehiscence, infection, and occasional lymphedema. Donor-site closure was most often achieved primarily; however, in a subset of cases, split- or full-thickness skin grafts were required to complete donor-site coverage. These values should be interpreted as descriptive estimates derived from aggregated data rather than statistically weighted pooled results, given the heterogeneity of study designs and outcome reporting.

Radiotherapy was reported across several included studies, reflecting its established role in the multidisciplinary treatment of extremity soft tissue sarcomas. Based on aggregated extractable data from studies reporting numerical radiotherapy information, neoadjuvant radiotherapy was reported in 33.5% of cases (65/194), while overall perioperative radiotherapy exposure was reported in 64.5% of cases (182/282). However, these denominators could not be reliably restricted to the propeller flap subgroup, because several studies reported radiotherapy data only for mixed cohorts.

Reporting of radiotherapy variables was inconsistent across studies, and detailed information regarding treatment timing or its relationship with reconstructive outcomes was often lacking. Consequently, radiotherapy-related data were summarized descriptively only, and no robust comparative inference could be drawn regarding its impact on reconstructive outcomes. Chemotherapy was less consistently reported and appeared limited to a minority of cases (Table 3).

Follow-up duration was variably reported but most commonly ranged between 12 and 24 months, with reported extremes from approximately 6 months to over 10 years. The estimated average follow-up was around 15–20 months. Oncologic outcomes were inconsistently reported across the included studies. Data on local recurrence were explicitly reported in only one of the nineteen included studies, which performed a comparative analysis between propeller flap and free flap reconstruction following extremity soft tissue sarcoma resection [42]. In this study, no statistically significant differences were observed in local recurrence-free survival or disease-free survival between the two reconstructive approaches. In other series, oncologic outcomes were only partially described. For example, Mallett et al. reported overall recurrence events during follow-up in a cohort of patients undergoing flap reconstruction after foot and ankle sarcoma resection, but without specific analysis according to reconstructive technique [44]. Similarly, Lafaye et al. reported the use of perioperative radiotherapy in a substantial proportion of patients but noted that detailed oncologic outcomes, including recurrence analysis, would be addressed in a separate study not yet available at the time of publication [47]. More broadly, studies focusing on sarcoma reconstruction emphasize the importance of adequate surgical margins and multidisciplinary management to achieve optimal oncologic outcomes, although they do not specifically analyze recurrence patterns according to flap type [3,51,52]. Overall, the available literature provides limited and heterogeneous information regarding oncologic endpoints, with most studies focusing primarily on reconstructive outcomes rather than long-term oncologic safety. Therefore, any inference regarding oncologic safety of propeller flaps in extremity sarcoma reconstruction should be interpreted cautiously, given the limited availability of recurrence-specific data. At present, the available evidence highlights a substantial gap in oncologic reporting rather than definitively addressing the question of oncologic safety.

Patient-reported outcomes were inconsistently documented but generally favorable. When available, both aesthetic and functional evaluations suggested high satisfaction and good functional recovery.

## 4. Discussion

Reconstruction after extremity soft tissue sarcoma resection remains particularly challenging, as it requires reliable soft-tissue coverage while preserving limb function and respecting oncologic principles. In this context, the present systematic review suggests that propeller flaps may represent a feasible reconstructive option in selected cases, although the available literature remains limited, largely based on small case series and case reports, and provides much stronger support for reconstructive feasibility than for any firm oncologic inference. Nevertheless, previous systematic analyses in non-oncologic contexts have demonstrated high flap survival rates and progressive adoption of propeller flaps over time, suggesting that their technical reliability may extend to oncologic reconstruction as well [40,53,54]. Importantly, the apparent size of the literature should not be overinterpreted. Although 656 patients were described across the included studies, the review question was ultimately informed by a much smaller subset of sarcoma-relevant propeller flap reconstructions. A key finding of this review is the predominance of mixed cohorts in the available literature. Many studies reporting propeller flaps include heterogeneous etiologies such as trauma, chronic wounds, burns, or other malignancies, making it difficult to isolate sarcoma-specific outcomes. This heterogeneity represents an important methodological limitation and highlights the need for more dedicated oncologic series. Among the few studies focusing specifically on sarcoma reconstruction, Cha et al. performed a comparative analysis evaluating patients undergoing extremity soft tissue sarcoma resection followed by adjuvant radiotherapy [42]. In their cohort, propeller flap reconstruction demonstrated local control and disease-free survival rates comparable to those observed in patients reconstructed with free flaps, with no statistically significant differences in oncologic outcomes.

From a theoretical perspective, however, propeller flaps raise concerns that differ from those associated with free tissue transfer. Unlike free flaps, which introduce tissue from distant donor sites, propeller flaps mobilize local tissues adjacent to the tumor bed. It has therefore been hypothesized that flap rotation could potentially alter the spatial relationship of surgical margins or complicate radiotherapy planning. Cha et al. specifically noted that rotation around the perforator pivot point may shift the surgical margin relative to the radiotherapy target volume [42]. Although the limited available literature has not identified a clear increase in recurrence among reported cases, it underscores the importance of multidisciplinary coordination between reconstructive surgeons and radiation oncologists. Consistent with these considerations, the adoption of propeller flaps in oncologic reconstruction appears to have occurred more cautiously than in elective or traumatic settings. While propeller flaps were rapidly integrated into reconstructive practice for non-oncologic indications, their use in sarcoma surgery likely expanded more gradually, reflecting both the technical learning curve of perforator-based techniques and initial concerns regarding oncologic safety. Importantly, the oncologic dimension of the available evidence remains substantially weaker than the reconstructive one. Recurrence-related outcomes were inconsistently reported across studies, and comparative data were extremely limited, precluding any robust inference regarding oncologic equivalence or safety.

Anatomically, the predominance of lower extremity reconstructions likely reflects both the epidemiology of soft tissue sarcomas and the reconstructive feasibility of propeller flaps in this region. Soft tissue sarcomas most frequently arise in the lower extremities, particularly in the thigh and peri-knee regions [3,55,56]. In addition, the lower limb often provides favorable conditions for propeller flap design due to predictable perforator anatomy and relative tissue laxity. Conversely, the relative scarcity of upper extremity cases may reflect both the lower incidence of sarcomas in this region and different reconstructive strategies, where limited local tissue availability and functional considerations often favor regional or microsurgical reconstruction.

**Figure 3:**
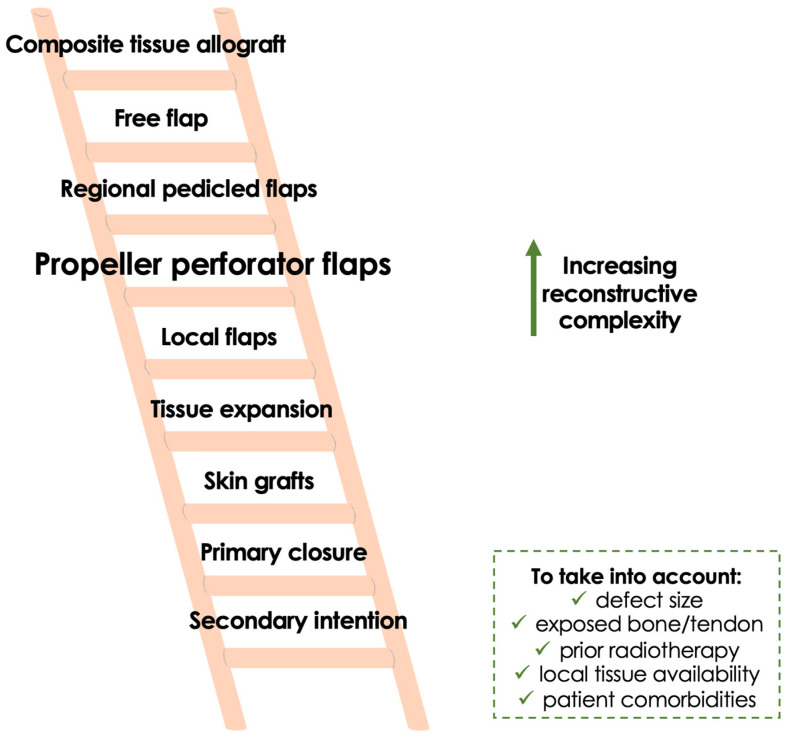
Updated reconstructive ladder for extremity sarcoma reconstruction. Propeller flaps represent an intermediate reconstructive option between traditional local flaps and microsurgical free tissue transfer. By allowing rotation of perforator-based local tissue, they expand the reconstructive possibilities for moderate-sized defects while avoiding the complexity of free flap reconstruction in selected patients.

From a reconstructive standpoint, the defects treated with propeller flaps in the included studies were generally moderate to large but rarely extremely extensive. This observation reflects the intrinsic limitations of local perforator-based reconstruction. Deep three-dimensional defects or composite resections involving bone, tendon, or neurovascular structures may exceed the reconstructive capacity of fasciocutaneous propeller flaps and continue to require microsurgical free tissue transfer. In this sense, propeller flaps appear to occupy an intermediate position within the reconstructive ladder, representing a “middle-ground” solution between traditional random local flaps and microsurgical free flaps (Figure 3).

Complication patterns across studies were relatively consistent, with venous congestion emerging as the most frequently reported mechanism of flap compromise [33,34,35]. This finding aligns with the known hemodynamic challenges associated with propeller flaps, particularly when large rotation arcs are required. Nevertheless, overall flap survival remained high across studies, supporting the technical reliability of the technique in carefully selected cases.

The interaction between propeller flaps and radiotherapy represents one of the most clinically relevant aspects of sarcoma reconstruction. Radiotherapy is frequently integrated into the multidisciplinary management of extremity soft tissue sarcomas, and a substantial proportion of patients undergoing reconstruction have received neoadjuvant or adjuvant treatment [4,56,57]. Reconstruction in irradiated fields presents well-known challenges, including impaired vascularity and compromised wound healing [58]. Some comparative observations suggest that pedicled perforator flaps may be associated with higher complication or reintervention rates in irradiated environments compared with free flaps [47,59]. However, other reports describe satisfactory outcomes with propeller flaps even in previously irradiated tissues, suggesting that outcomes may depend on patient selection, perforator quality, and surgical expertise [32,50].

Another relevant aspect is donor-site morbidity, which is often underreported. Although propeller flaps avoid the donor-site morbidity associated with free tissue transfer, closure of large local defects may still require skin grafting or lead to functional limitations, particularly in limbs already compromised by oncologic resection or radiotherapy. In addition, successful outcomes appear closely related to surgeon experience and preoperative perforator mapping. The increasing use of Doppler ultrasonography or CT angiography for perforator identification may further improve flap planning and reliability [33].

Several limitations should be acknowledged. First, the available evidence is largely based on small retrospective studies, case series, and case reports, limiting the strength of the conclusions. Second, several included studies were not exclusively focused on sarcoma reconstruction but reported mixed oncologic or non-oncologic populations, requiring selective extraction of sarcoma-related propeller flap data. This process, while necessary, further limits the strength and specificity of the available evidence. Third, follow-up duration was variable and often limited, particularly with respect to oncologic endpoints. Fourth, outcome definitions and reporting were inconsistent across studies, reducing comparability between series and limiting interpretation of both reconstructive and oncologic outcomes. Fifth, publication and indication biases are likely present, as propeller flaps may preferentially be reported in selected or successful cases. In addition, the operator-dependent nature of propeller flap surgery suggests that outcomes may partially reflect institutional expertise, while geographic clustering of studies may indicate regional differences in reconstructive philosophy. An additional limitation is the inconsistent reporting of tumor depth across the included studies. The distinction between superficial and deep soft tissue sarcomas may have influenced both oncologic management, including the indication for perioperative radiotherapy, and reconstructive strategy. In particular, a greater proportion of superficial lesions may partly explain the lower reported use of radiotherapy in some series and the relatively infrequent need for flaps including a muscular component. However, this variable was not reported consistently enough to allow meaningful analysis. Despite these limitations, the available evidence suggests that propeller flaps represent a viable reconstructive option in selected cases of extremity sarcoma. Rather than replacing free flaps, they appear to complement the reconstructive armamentarium by offering a reliable local alternative in appropriately selected patients. Future prospective studies with standardized reporting of oncologic, functional, and quality-of-life outcomes are needed to better define their role within modern sarcoma reconstruction.

## 5. Conclusions

This systematic review highlights the evolving role of propeller flaps in extremity sarcoma reconstruction. Although the available evidence remains limited and largely based on small retrospective series, the current literature suggests that propeller flaps can achieve reliable reconstructive outcomes with high survival rates when applied in carefully selected patients. From an oncologic perspective, the currently available literature does not identify a clear signal of increased local recurrence in selected reported cases; however, oncologic outcomes are too limited and inconsistently reported to support definitive conclusions regarding safety or equivalence with alternative reconstructive strategies. While theoretical concerns remain regarding local tissue mobilization and the proximity of reconstructed tissues to the tumor bed, the currently available evidence does not yet allow robust comparison with alternative reconstructive strategies. Therefore, these findings should be interpreted cautiously in light of the heterogeneity and methodological limitations of the existing literature.

Rather than replacing microsurgical free flaps, propeller flaps may occupy a distinct reconstructive “middle-ground” in selected cases, bridging the gap between traditional local flaps and free tissue transfer. Their role appears most relevant when local perforator-based tissue transfer can provide adequate coverage without clearly requiring microsurgical reconstruction, offering a pragmatic balance between surgical simplicity and reconstructive reliability [60].

## Figures and Tables

**Table 1:** Characteristics and reconstructive outcomes of the included studies. Data are reported at the flap level when available. NR = not reported; NA = not available for the sarcoma subgroup; STS = soft tissue sarcoma.

Author (Year)	Study Design	Study Quality Assessment	Patients (*n*)	Limb Location	Histology (If Reported)	Propeller Flaps After Sarcoma Excision (*n*)	Flap Survival (*n*)	Total Flap Loss	Partial Necrosis	Other Complications
Lecours (2010) [32]	Retrospective	Good	49	Lower limb	Mixed STS	6	4	0	2	No
Higueras Suñé (2011) [33]	Retrospective	Good	18	Lower limb	Leiomyosarcoma	1 specifically reported after sarcoma excision	15	0	3	1 venous congestion
Tos (2011) [34]	Retrospective	Good	22	Lower limb	Mixed STS	6	4	0	2	1 venous congestion spontaneously resolved
Shin (2012) [35]	Retrospective	Good	8	Lower limb	NR	2	2	0	0	2 venous congestion spontaneously resolved
Cadanelli (2015) [36]	Case report	Good	1	Lower limb	NR	1	1	0	0	No
Nambi (2015) [37]	Case report	Fair	1	Lower limb	NR	1	1	0	0	No
Zang (2015) [38]	Case report	Fair	2	Upper limb	Spindle cells sarcoma	1	1	0	0	No
Feng (2016) [39]	Prospective comparative analysis	Good	40	Lower limb	Spindle cells sarcoma	1 specifically reported after sarcoma excision	1	0	0	No
Innocenti (2019) [40]	Retrospective	Good	179	Lower limb	NR	21	21	NA	NA	NA
Yoshida (2019) [41]	Case report	Good	1	Lower limb	NR	2	2	0	0	1 lymphedema
Cha (2020) [42]	Retrospective	Good	88	Lower limb	Mixed STS	32	NR	NR	NR	NR
Brunetti (2021) [43]	Retrospective	Good	24	Lower limb	NR	10	10	0	0	1 wound dehiscence
Mallett (2021) [44]	Retrospective	Good	44	Lower limb	Mixed STS	10	9	1	0	0
Zhu (2021) [45]	Retrospective	Good	29	Lower limb	NR	6	4	1	1	0
Benedict (2022) [46]	Case series	Good	3	Lower limb	Mixed STS	3	3	0	0	0
Lafaye (2022) [47]	Retrospective	Good	74	Lower limb	Mixed STS	51	49	NA	NA	NA
Tada (2022) [48]	Case report	Good	2	Lower limb	Mixed STS	2	2	0	0	0
Alban (2024) [49]	Case report	Good	1	Lower limb	Myxofibrosarcoma	1	1	0	0	1 wound dehiscence
Brunetti (2024) [50]	Retrospective	Good	70	Lower limb	Mixed STS	28	NA	NA	NA	NA

**Table 2:** Summary of the main findings reported across the included studies. Values represent descriptive estimates based on the available data reported in the literature. Percentages were calculated from aggregated extractable data and represent descriptive estimates only.

Variable	Summary of Findings
Included studies	19
Total patients reported	656
Patients reconstructed with propeller flaps	185
Mean patient age	59.8 years (range 11–92)
Sex distribution	Slight female predominance (M:F ≈ 0.87)
Most common reconstruction site	Lower extremity (>95% of cases)
Upper extremity reconstruction	Rare (≈2–5% of cases)
Typical defect size	Moderate-to-large defects, commonly 10–15 cm in maximal diameter
Flap characteristics	Predominantly fasciocutaneous propeller flaps elevated in a subfascial plane
Rotation arc	Usually between 90° and 180°
Flap survival (extractable flap-level data)	92.7%
Total flap loss (extractable flap-level data)	3.8%
Partial flap necrosis (extractable flap-level data)	9.6%
Most frequently reported complication	Venous congestion
Donor-site closure	Usually primary closure; skin graft occasionally required
Radiotherapy exposure	Frequently reported but inconsistently quantified
Follow-up duration	Variable; most commonly between 12 and 24 months
Patient-reported outcomes	Generally favorable when reported, but inconsistently assessed

**Table 3:** Radiotherapy exposure and oncologic context in the included studies. Findings are summarized descriptively due to heterogeneous reporting of oncologic treatment and reconstructive outcomes across studies.

Variable	Summary of Findings
Neoadjuvant radiotherapy	33.5% (65/194) based on aggregated extractable data from studies reporting numerical values
Overall perioperative radiotherapy	64.5% (182/282) based on aggregated extractable data from studies reporting numerical values
Chemotherapy use	Infrequently reported and inconsistently documented across studies
Reconstruction in irradiated fields	Reported in multiple series, indicating that propeller flaps are sometimes performed in previously irradiated tissues
Impact of radiotherapy on reconstructive outcomes	Evidence heterogeneous; some studies suggest higher complication or reintervention rates in irradiated fields
Comparative observations	One comparative series reported higher reintervention rates in irradiated defects reconstructed with pedicled flaps compared with free flaps
Oncologic outcomes in irradiated patients	Limited evidence available; no clear difference in recurrence rates compared with free flap reconstruction reported in the only comparative study

## Data Availability

No data collection forms, extracted data, analytic code or other materials used in this review are publicly available, but are available from the corresponding author upon reasonable request. This systematic review was registered in PROSPERO (registration number: CRD420261349550).

## Supplementary Materials

The following supporting information can be downloaded at https://www.mdpi.com/article/10.3390/curroncol33050269/s1, File S1: Electronic search strategies; Table S1: PRISMA checklist.
