# Supplementary material for: Propeller Flaps in Extremity Sarcoma Reconstruction: A Systematic Review of Reconstructive Outcomes and Oncologic Considerations
Source: Curr Oncol. 2026 May 6;33(5):269. doi: 10.3390/curroncol33050269 (PMC13206663; doi:10.3390/curroncol33050269)
Supplement: Supplementary file 1 [file curroncol-33-00269-s001.zip › File S1.pdf]

Electronic search strategies

The search strategy was adapted for each database according to its specific indexing system.

PubMed/MEDLINE

("propeller flap"[tiab] OR "propeller flaps"[tiab] OR "perforator flap"[tiab] OR "perforator flaps"[tiab] OR "perforator based flap"[tiab] OR "perforator-based flap"[tiab] OR "perforator-based flaps"[tiab] OR "perforator based flaps"[tiab] OR "rotation flap"[tiab] OR "rotation flaps"[tiab] OR "local flap"[tiab] OR "local flaps"[tiab] OR "fasciocutaneous flap"[tiab] OR "fasciocutaneous flaps"[tiab] OR "axis flap"[tiab] OR "axis flaps"[tiab] OR "propeller perforator"[tiab] OR "perforator propeller"[tiab] OR "peroneal perforator"[tiab] OR "peroneal artery perforator"[tiab] OR "tibial perforator"[tiab] OR "posterior tibial perforator"[tiab] OR "anterior tibial perforator"[tiab] OR "radial perforator"[tiab] OR "radial artery perforator"[tiab] OR perforator[tiab] OR propeller[tiab])
AND
("sarcoma"[tiab] OR "soft tissue sarcoma"[tiab] OR "soft tissue neoplasm"[tiab] OR "soft tissue tumor"[tiab] OR "soft tissue tumour"[tiab] OR "STS"[tiab] OR "malignant tumor"[tiab] OR "malignant tumour"[tiab] OR "limb salvage"[tiab] OR "limb-sparing"[tiab])
AND
("limb"[tiab] OR "extremity"[tiab] OR "lower limb"[tiab] OR "upper limb"[tiab] OR "leg"[tiab] OR "arm"[tiab] OR "hand"[tiab] OR "foot"[tiab] OR "thigh"[tiab] OR "forearm"[tiab] OR "ankle"[tiab])

Scopus

TITLE-ABS-KEY (("propeller flap" OR "propeller flaps" OR "perforator flap" OR "perforator flaps" OR "tibial perforator" OR "peroneal perforator" OR "radial perforator")
AND
("soft tissue sarcoma" OR sarcoma)
AND
(leg OR arm OR foot OR hand OR thigh OR forearm OR "lower limb" OR "upper limb"))

Cochrane Library

("propeller flap" OR "propeller flaps" OR "perforator flap")
AND
("soft tissue sarcoma" OR sarcoma)
AND
(limb OR extremity)
